# Trimethylamine N-oxide predicts cardiovascular events in coronary artery disease patients with diabetes mellitus: a prospective cohort study

**DOI:** 10.3389/fendo.2024.1360861

**Published:** 2024-07-18

**Authors:** Xue Yu, Yijia Wang, Ruiyue Yang, Zhe Wang, Xinyue Wang, Siming Wang, Wenduo Zhang, Jun Dong, Wenxiang Chen, Fusui Ji, Wei Gao

**Affiliations:** ^1^ Department of Cardiology and Institute of Vascular Medicine, Peking University Third Hospital, Beijing, China; ^2^ Department of Cardiology, Beijing Hospital, National Center of Gerontology, Institute of Geriatric Medicine, Chinese Academy of Medical Sciences, Beijing, China; ^3^ Fuwai Hospital, Chinese Academy of Medical Sciences & Peking Union Medical College/National Center for Cardiovascular Diseases, Beijing, China; ^4^ The Key Laboratory of Geriatrics, Beijing Institute of Geriatric Medicine, Chinese Academy of Medical Sciences, Beijing Hospital/National Center of Gerontology of National Health Commission, Beijing, China; ^5^ Department of Cardiology, China-Japan Friendship Hospital (Institute of Clinical Medical Sciences), Chinese Academy of Medical Sciences & Peking Union Medical College, Beijing, China; ^6^ State Key Laboratory of Vascular Homeostasis and Remodeling, Peking University; NHC Key Laboratory of Cardiovascular Molecular Biology and Regulatory Peptides, Peking University; Beijing Key Laboratory of Cardiovascular Receptors Research, Beijing, China

**Keywords:** trimethylamine N-oxide, coronary artery disease, diabetes mellitus, major adverse cardiovascular events, prospective cohort study

## Abstract

**Background:**

Gut microbiota has significant impact on the cardio-metabolism and inflammation, and is implicated in the pathogenesis and progression of atherosclerosis. However, the long-term prospective association between trimethylamine N-oxide (TMAO) level and major adverse clinical events (MACEs) in patients with coronary artery disease (CAD) with or without diabetes mellitus (DM) habitus remains to be investigated.

**Methods:**

This prospective, single-center cohort study enrolled 2090 hospitalized CAD patients confirmed by angiography at Beijing Hospital from 2017-2020. TMAO levels were performed using liquid chromatography-tandem mass spectrometry. The composite outcome of MACEs was identified by clinic visits or interviews annually. Multivariate Cox regression analysis, Kaplan-Meier analysis, and restricted cubic splines were mainly used to explore the relationship between TMAO levels and MACEs based on diabetes mellitus (DM) habitus.

**Results:**

During the median follow-up period of 54 (41, 68) months, 266 (12.7%) developed MACEs. Higher TMAO levels, using the tertile cut-off value of 318.28 ng/mL, were significantly found to be positive dose-independent for developing MACEs, especially in patients with DM (HR 1.744, 95%CI 1.084-2.808, *p* = 0.022).

**Conclusions:**

Higher levels of TMAO are significantly associated with long-term MACEs among CAD patients with DM. The combination of TMAO in patients with CAD and DM is beneficial for risk stratification and prognosis.

## 1 Introduction

Coronary artery disease (CAD) is a prevalent and escalating public health concern that significantly contributes to the global burden of morbidity and mortality (1). Diabetes mellitus (DM) is recognized as major risk factor for cardiovascular complications (2). Cardiovascular events are the primary cause of morbidity and mortality in individuals with diabetes, who have a twofold higher risk of cardiovascular events than non-diabetic individuals (3–5). Early detection and management of these risk factors may significantly reduce the incidence or delay the onset of major adverse clinical events (MACEs) and improve our understanding of the pathophysiology of CAD (6).

Previous publications had identified that the gut microbiota as a novel contributor to the progression of cardiovascular disease (CVD) and DM, which transforms dietary choline, betaine, and carnitine into trimethylamine (TMA) (7). Subsequently, the trimethylamine N-oxide (TMAO) were transformed from TMA oxidation by flavin-containing monooxygenase-3 (FMO3) in the liver (8).Preclinical studies have elucidated the role of TMAO elucidated the role of TMAO in disrupting glucose and lipid homeostasis, leading to glucose intolerance, insulin resistance, and oxidative stress in adipose tissue (9, 10). Plasma TMAO has been associated with cardiovascular disease (CVD) in clinical and population-based studies, with further evidence indicating proatherogenic and prothrombotic functions (11, 12). However, the predictive value of TMAO levels in patients suffering from coronary artery disease with and without diabetes mellitus has not been clarified. The objective of our study is to explore the association between plasma TMAO levels and the incidence of MACEs among participants in the Beijing Hospital Atherosclerosis Study (BHAS), stratified by the presence of DM.

## 2 Materials and methods

### 2.1 Protocol design and populations

The BHAS study is a prospective, single-center, observational cohort study, which included 2970 coronary angiographic patients complained for chest pain in Beijing Hospital from March 2017 to March 2020. The exclusion criteria were as follows: (I) have undergone an aortic dissection, have a pulmonary embolism, malignant tumor, autoimmune disorder, severe infectious disease, or trauma, have undergone a recent surgical procedure, have had severe heart failure with a left ventricular ejection fraction <20%, have liver dysfunction [alanine amino transferase (ALT) level >135 U/L], severe renal dysfunction [creatinine (Cr) >4.0 mg/dL], or blood-borne infectious diseases, including human immune-deficiency virus/acquired immunodeficiency syndrome, hepatitis B, or hepatitis C; (II) have a significant malignancy or tumor disease that will require advanced medical or surgical therapy or both in the following year; (III) have other major systemic diseases that will require hospitalization or operation in the following year; (IV) are unable or unwilling to be followed up during the subsequent 1-year period; (V) have a life expectancy of <12 months; (VI) are unable to complete the baseline questionnaires; and/or (VII) are pregnant or nursing, suffering from alcoholism or drug abuse, or have a mental illness for which they are undergoing treatment (13). After recruitment, all patients were given a detailed written consent form explaining the study's objectives, duration, and specimen collection. This study was screened and approved by the Beijing Hospital Ethics Committee (2016BJYYEC-121-02) and registered at ClinicalTrials.gov, NCT03072797. This study followed the Strengthening the Reporting of Observational Studies in Epidemiology (STROBE) reporting guideline for cohort studies.

### 2.2 Clinical and laboratory data

We collected demographic and medical history data such as sex, age, height, weight, and blood pressure; history of DM, hypertension (HTN), dyslipidemia, and stroke; family history of premature CAD; and current smoking status. Blood samples were collected in the morning after an overnight fast using regular vacuum tubes. The serum was separated within two hours, and aliquots were coded and stored at -80°C until analysis. Blood was obtained by puncture of the radial artery prior to CAG and TMAO measurements were performed using liquid chromatography-tandem mass spectrometry. Liquid chromatography separation is performed using an Agilent 1260 Series High-performance liquid chromatography system (Santa Clara, CA, USA). Tandem mass spectrometry (MS)/MS detection is carried out on an AB Sciex 5500 QTRAP system (Framingham, MA, USA) with both positive and negative electronic spray ionization in multiple-reaction monitor mode. All patient samples will be analyzed, with 88 samples in each run. Additionally, 2 quality controls materials will be measured in each run to monitor inter-run variations. The quantification was carried out by the standard curve internal standard method, and the concentration of TMAO in the standard solution was the x-axis, and the corresponding ratio of TMAO to the peak area of the internal standard was the y-axis, and linear regression was carried out. The peak area ratio of TMAO to the internal standard in each sample was brought into the regression equation to calculate the TMAO concentration (13). Other laboratory assays were evaluated in the clinical laboratory of Beijing Hospital using assay kits from Sekisui Medical Technologies (Osaka, Japan) on a Hitachi 7180 chemistry analyzer.

### 2.3 Diagnosis of CAD

The diagnosis of CAD was established utilizing the patient’s electronic medical records, which primarily involved the examination of coronary angiograms. These angiograms were obtained using standard techniques and recorded by two experienced interventional cardiologists from multiple angles. The built-in QCA software of the Allura Per FD20 Angiography System (Philips Healthcare, Netherlands) analyzed all targeted coronary lesions of the patients. CAD was defined as the presence of stenosis of 50% or higher in diameter in any of the coronary arteries or major branches. Non-CAD subjects had stenosis of less than 50% or negative findings in computed tomography of the coronary arteries or stress myocardial perfusion imaging.

### 2.4 Outcome and follow-up

The endpoint was the composite outcome of MACEs, which included nonfatal myocardial infarction, stroke, and all-cause mortality. The diagnosis of myocardial infarction (MI) was based on the fourth universal definition, which included symptoms of ischemia and either ST-segment shifts on electrocardiography or elevated troponin levels. Ischemic stroke was diagnosed by confirming an acute neurological deficit through computed tomography or magnetic resonance imaging. Patients were followed up by clinic revisit or telephone interviews annually, and adverse incidents were recorded during each visit.

### 2.5 Statistical analysis

Continuous variables were expressed with means or medians, and compared using Student t-tests or Mann-Whitney U tests. Categorical variables are expressed as numbers/percentages and compared with the chi-square test. We used both univariate and multivariate Cox proportional hazard models to investigate the connection between TMAO and MACEs. To determine if the link between TMAO and MACEs was statistically significant, we calculated the hazard ratio (HR) based on the TMAO concentration decile. Additionally, we conducted Kaplan–Meier analysis to determine the time to MACE, accounting for the tertile of TMAO concentrations for DM and non-DM patients (371.59 ng/mL and 265.28 ng/mL, respectively). We compared the survival curves using the log-rank test. To flexibly model the relationship between TMAO levels and MACEs in patients with and without DM, we utilized restricted cubic splines (RCS) with four knots at the 5th, 25th, 75th, and 95th centiles. The tertile concentrations of TMAO (371.59 ng/mL and 265.28 ng/mL, respectively) served as the reference. Model 1 was the base design and did not adjust for any confounders. The effects of age, gender, BMI, current smoking on outcomes were considered in Model 2. Model 3 builds on model 2 by considering the following confounders: hypertension, hyperlipidemia, DM, stroke, family history of premature CAD, prior PCI, Creatinine, ALT, AST, revascularization. All statistical analyses were conducted using SPSS software (version 26.0, SPSS Inc., Chicago, IL, USA) and R (version 4.0.3). Two-tailed with *p*-values < 0.05 were considered statistically significant.

## 3 Results

### 3.1 Baseline characteristics

**Figure 1:**
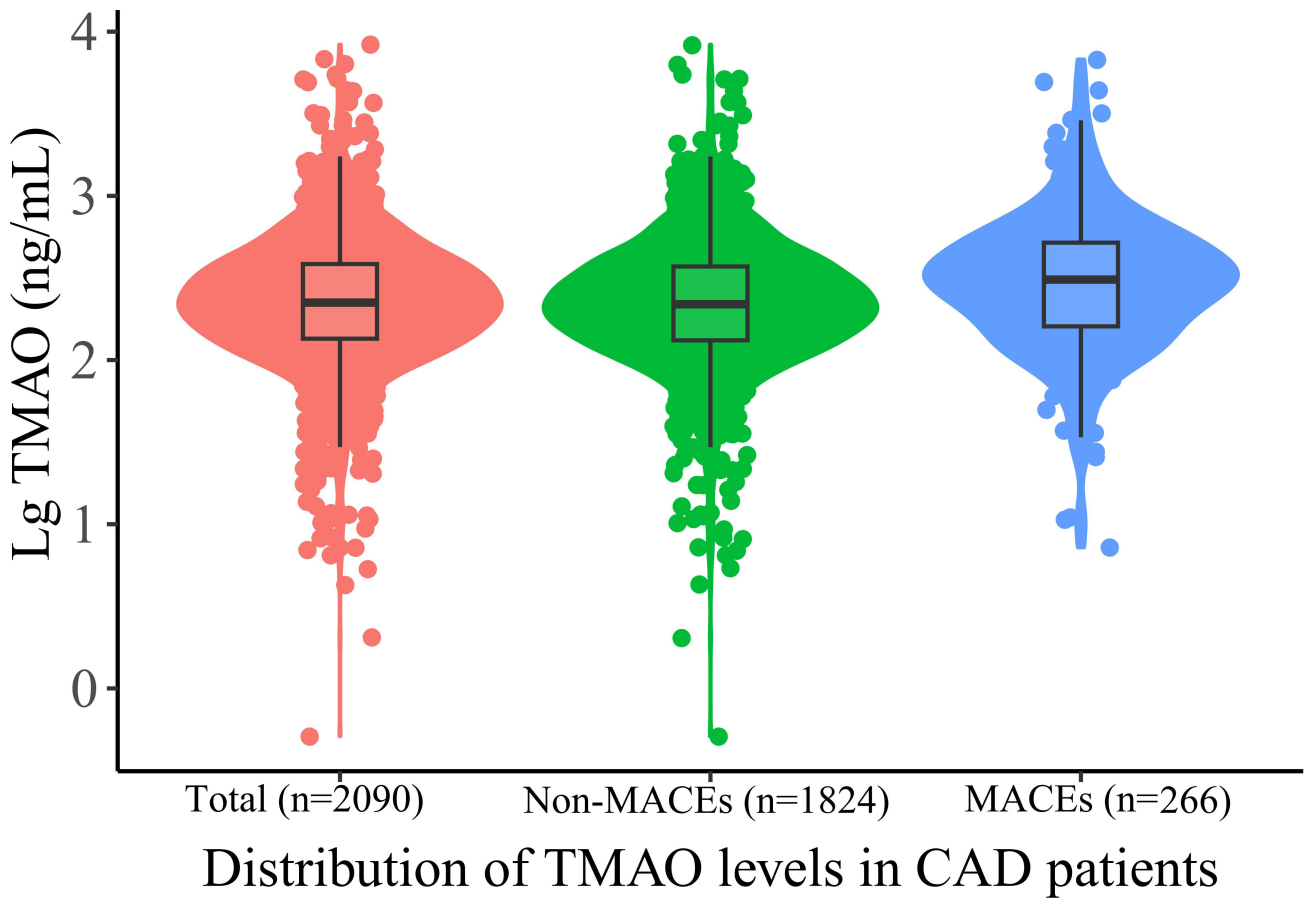
Violin plots of the distribution of TMAO levels in CAD patients. Different color dots represent measured TMAO levels in different groups; The top of the box: 75th percentile; The horizontal line: 50th percentile (median); The bottom of the box: 25th percentile; A line represents the distribution area of the 95% confidence interval of the data. TAMO, trimethylamine N-oxide; MACEs, major adverse cardiovascular events.

Among the total of 2970 patients undergoing coronary angiography in BHAS, 289 CAD patients lost follow-up, and 2090 patients ultimately entered this study and completed the follow-up process, as shown in **Supplementary Figure 1**. The mean age of the patients was 66.2 years, and 66.6% being male, with 52.3% being DM. The patients were divided into three groups based on their TMAO levels:the low TMAO group (Tertile 1 group), the middle TMAO group (Tertile 2 group), and the high TMAO group (Tertile 3 group). In **Table 1**, the CAD patients in Tertile 3 group were more older, more likely to be with hypertension, DM, and a history of stroke. More individuals are taking angiotensin converting enzyme inhibitors or angiotensin receptor blocker. Additionally, CAD patients in the high TMAO group exhibit higher levels of blood glucose, creatinine. The patients in the MACEs group were older and more likely to have diabetes mellitus, and a history of stroke, and higher creatine levels and TMAO levels (**Supplementary Table 1**). The median concentration of TMAO was higher in the MACEs group than in the non-MACEs group [302.61 (157.54-515.40) ng/mL vs. 215.53 (129.22-371.84) ng/mL, *p* < 0.001]. As shown in **Figure 1**, To provide a more intuitive representation of the differences in TMAO levels between groups, we took the logarithm of TMAO levels as the vertical axis and created violin plots to display the TMAO levels in the overall population, MACEs group, and non-MACEs group. In **Supplementary Table 2**, patients with DM had significantly higher levels of TMAO than patients without DM [192.66 (122.54, 307.68) ng/mL vs. 264.89 (144.66, 442.40) ng/mL, respectively, *p <*0.001].

**Table 1:** Baseline demographics and clinical characteristics.

Variables	All (n=2090)	Tertile 1 (n=698)	Tertile 2 (n=696)	Tertile 3 (n=696)	*p-*value
age, years	66.2 ± 10.8	62.8 ± 10.5	66.4 ± 10.0	69.4 ± 10.8	<0.001
male, %	1392(66.6%)	466(66.8%)	490(70.4%)	436(62.6%)	0.009
BMI, kg/m^2^	26.2 ± 6.7	26.2 ± 6.8	26.1 ± 5.8	26.1 ± 7.3	0.973
current smoking, %	703(33.6%)	239(34.2%)	253(36.4%)	211(30.3%)	0.051
hypertension, %	1654(79.1%)	521(74.6%)	540(77.6%)	593(85.2)	<0.001
hyperlipidemia, %	976(46.7%)	346(49.6%)	316(45.4%)	314(45.1%)	0.257
DM, %	1094(52.3%)	310(44.4%)	335(48.1%)	449(64.5%)	<0.001
stroke, %	241(11.5%)	57(8.2%)	91(13.1%)	93(13.4%)	0.003
history of statin use, %	773(37.0%)	269(38.5%)	271(38.9%)	233(33.5%)	0.202
family history of premature CAD, %	164(7.8%)	65(9.3%)	57(8.2%)	42(6.0%)	0.061
prior PCI, %	801(38.3%)	250(35.8%)	269(38.6%)	282(40.5%)	0.367
Laboratory test					
TC, mmol/L	3.54 ± 0.80	3.59 ± 0.82	3.51 ± 0.78	3.52 ± 0.80	0.142
TG, mmol/L	1.77 ± 0.97	1.76 ± 1.10	1.73 ± 0.93	1.79 ± 0.88	0.528
HDL-C, mmol/L	1.00 ± 0.26	1.01 ± 0.26	1.00 ± 0.26	0.98 ± 0.25	0.067
LDL-C, mmol/L	2.02 ± 0.70	2.07 ± 0.72	2.00 ± 0.69	2.00 ± 0.69	0.132
FBG, mmol/L	7.1 ± 2.7	6.71 ± 2.42	7.02 ± 0.75	7.53 ± 2.90	<0.001
ALT	19.60(14.80,27.70)	20.90(15.48,29.83)	18.80(14.80,27.20)	18.70(13.90,26.28)	<0.001
AST	20.40(17.60,25.10)	20.95(17.70,25.40)	19.90(17.50,25.30)	20.40(17.50,24.70)	0.157
Crea,umol/L	78.00(68.00,89.00)	75.00(66.00,83.00)	79.00(68.00,88.00)	82.00(70.00,100.75)	<0.001
Medication at discharge					
Aspirin,%	1831(87.6%)	616(88.3%)	611(87.8%)	604(86.8%)	0.171
Clopidogrel,%	1435(68.7%)	472(67.6%)	476(68.4%)	487(70.0%)	0.125
Ticagrelor,%	41(2.0%)	16(2.3%)	16(2.3%)	9(1.3%)	0.078
ACEI/ARB, %	886(42.4%)	267(38.3%)	288(41.4%)	331(47.6%)	<0.001
β-bloker, %	1176(56.3%)	391(56.0%)	394(56.6%)	391(56.2%)	0.194

BMI, body mass index; DM, diabetes mellitus; CAD, coronary artery disease; PCI, percutaneous coronary intervention; TC,total cholesterol; TG,triglycerides; HDL-C, high-density lipoprotein cholesterol; LDL-C, low-density lipoprotein cholesterol; ALT, alanine aminotransferase; AST, aspartate aminotransferase; TMAO, trimethylamine N-oxide; ACEI, angiotensin converting enzyme inhibitors; ARB, angiotensin receptor blocker.

### 3.2 The impact of high TMAO levels on MACEs in the overall cohort

**Figure 2:**
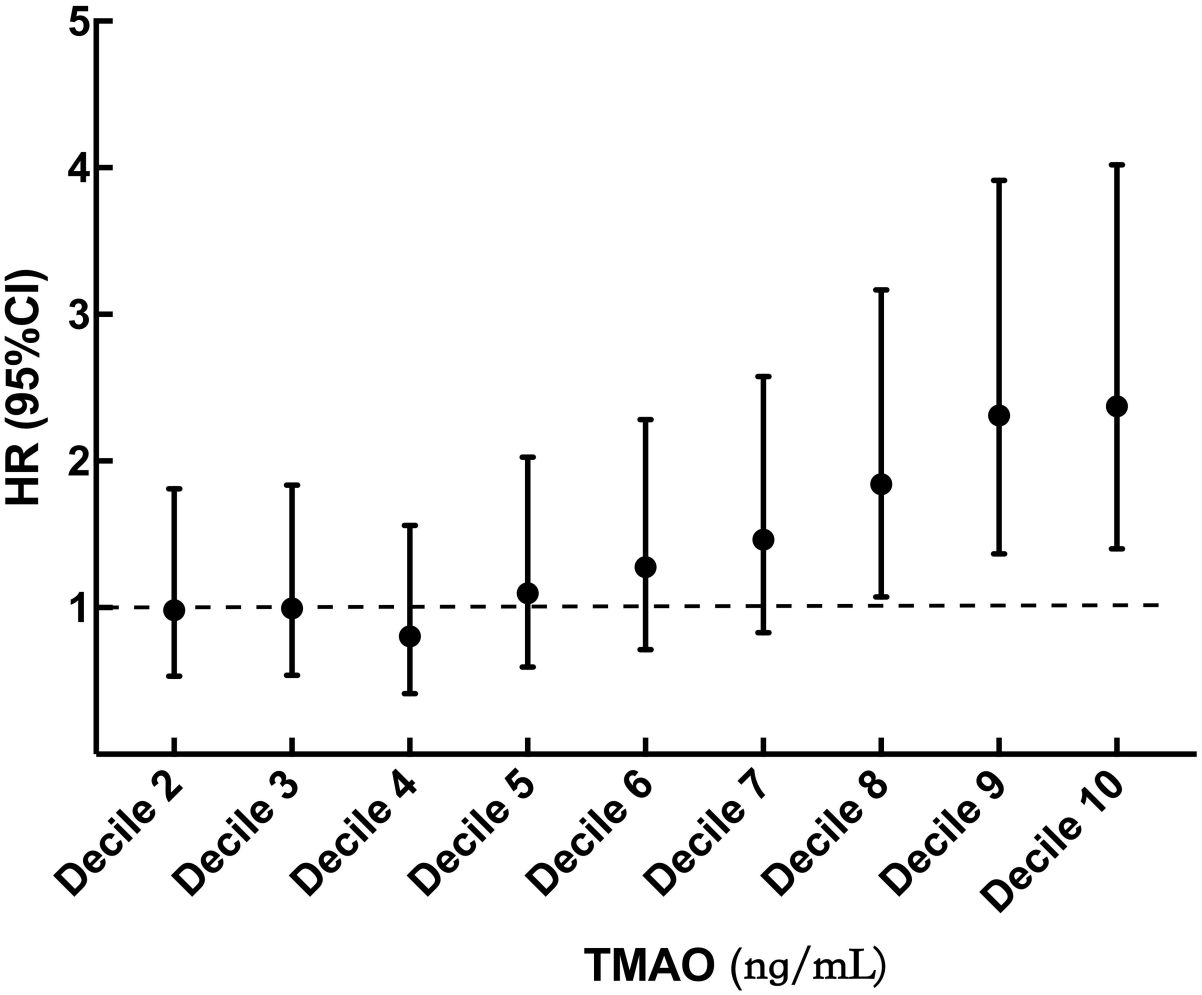
Hazard ratio by decile of TMAO. TMAO, trimethylamine N-oxide. Filled circles and vertical lines indicate the hazard ratio and 95% CI for deciles 2 to 10 of TMAO relative to decile 1. Decile 1 (TMAO < 76.91 ng/mL); decile 2 (76.91 ≤ TMAO < 115.59 ng/mL); decile 3 (115.59 ≤ TMAO < 149.87 ng/mL); decile 4 (149.87 ≤ TMAO < 184.70 ng/mL); decile 5 (184.70 ≤ TMAO < 223.83 ng/mL); decile 6 (223.83 ≤ TMAO < 274.38 ng/mL); decile 7 (274.38 ≤ TMAO < 342. 18 ng/mL); decile 8 (342.18 ≤ TMAO < 438.00 ng/mL); decile 9 (438.00 ≤ TMAO < 642.80 ng/mL); decile 10 (TMAO ≥ 642.80 ng/mL).

During median follow-up of 54 (41, 68) levemonths, 266 (12.7%) patients reached MACEs, including 152(57.1%)deaths, 48(18.0%)nonfatal myocardial infarctions, and 66(24.8%)strokes. To analyze the effect of different levels of TMAO on MACEs outcomes, TMAO levels were analyzed in deciles. Patients with TMAOls above decile 8 (TMAO≥342.18 ng/mL) had a significantly higher risk of MACEs than those in the first decile. The risk increased in patients in decile 8 (HR 1.843, 95% CI: 1.073-3. 167, *p* = 0.027), decile 9 (HR 2.312, 95% CI: 1.366-3.914, *p* = 0.002), and decile 10 (HR 2.373, 95% CI: 1.401-4.019, *p* = 0.001) (**Figure 2**). In **Table 2**, multivariate Cox analysis showed that TMAO levels in the tertile 3 group, the MACEs risk increased by 47.7% more than that in the tertile 1 group (HR=1.474, 95% CI: 1.079- 2.022, *p* = 0.015).

**Table 2:** Relationship between MACEs and TMAO Levels, stratified by DM, in the multivariate Cox regression analysis model.

Variables		Model 1		Model 2		Model 3	
		*HR*(95%*CI*)	*p*-value	*HR*(95%*CI*)	*p*-value	*HR*(95%*CI*)	*p*-value
CAD (n=2090)	Tertile 1	/	/	/	/	/	/
	Tertile 2	1.062(0.760-1.485)	0.723	0.946(0.675-1.326)	0.747	0.926(0.660-1.300)	0.657
	Tertile 3	2.099(1.566-2.813)	<0.001	1.666(1.228-2.260)	0.001	1.477(1.079-2.022)	0.015
CAD-DM (n=1094)	Tertile 1	/	/	/	/	/	/
	Tertile 2	1.088(0.673-1.757)	0.732	0.996(0.612-1.620)	0.986	0.917(0.561-1.498)	0.730
	Tertile 3	2.216(1.417-3.467)	<0.001	1.944(1.219-3.101)	0.005	1.744(1.084-2.808)	0.022
CAD-non-DM (n=996)	Tertile 1	/	/	/	/	/	/
	Tertile 2	1.016(0.636-1.624)	0.947	0.918(0.574-1.469)	0.722	0.916(0.570-1.474)	0.719
	Tertile 3	1.889(1.269-2.811)	0.002	1.426(0.944-2.153)	0.092	1.306(0.857-1.989)	0.214

Model1:crude risk; Model2:adjusted age, gender, BMI, current smoking; Model3:variables in model 2 plus hypertension, hyperlipidemia, DM, stroke, family history of premature CAD, prior PCI, Creatinine, ALT, AST, revascularization.

MACE, major adverse cardiovascular event; DM, diabetes mellitus; TMAO, trimethylamine N-oxide; CAD, coronary artery disease; HR, hazard ratio; CI, Confidence interval; BMI, Body mass index; PCI, percutaneous coronary intervention; ALT, alanine aminotransferase; AST, aspartate aminotransferase.

### 3.3 Analysis of the nonlinear relationship between TMAO and MACEs

**Figure 3:**
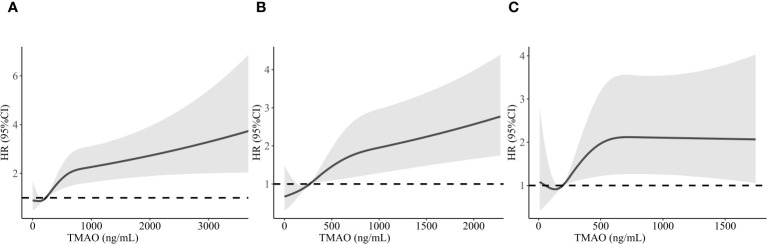
Restricted cubic spline analysis of MACEs risk as a function of TMAO concentration in CAD patients **(A)**, CAD with DM patients **(B)**, and CAD with non-DM patients **(C)**. MACEs, major adverse cardiovascular events; TMAO, trimethylamine N-oxide; CAD, coronary artery disease.

We examined the connection between TMAO concentrations and the likelihood of MACEs in CAD patients using restricted cubic splines. As demonstrated in **Figure 3A**, the association was not linear across the entire distribution; we observed a threshold effect, with apparent dose–response associations at higher levels. The risk of MACEs was relatively flat until approximately 318.28 ng/mL TMAO and then increased rapidly afterwards (*p* for nonlinearity <0.001). For patients with DM, we found that the risk of MACEs increased with increasing TMAO concentrations, reaching 371.59 ng/mL (*p* for nonlinearity = 0.023), as shown in **Figure 3B**. For non-DM patients, the risk of MACEs increased progressively with increasing TMAO concentrations above 265.28 ng/mL (*p* for nonlinearity = 0.026), as shown in **Figure 3C**.

### 3.4 The role of TMAO in predicting MACEs in CAD patients with and without DM

**Figure 4:**
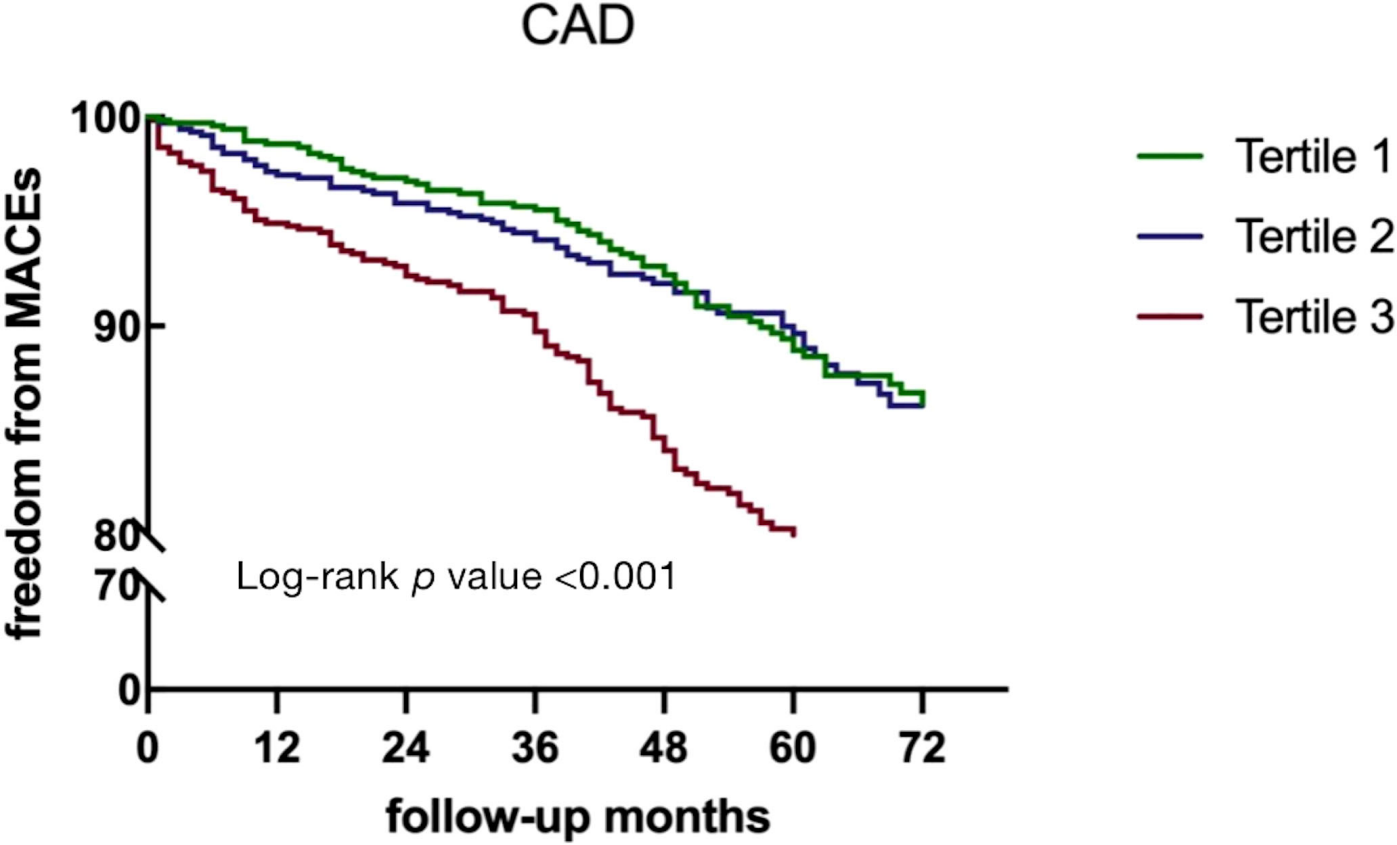
Kaplan–Meier curves for MACEs according to TMAO levels in CAD patients. DM, diabetes mellitus; TMAO, trimethylamine N-oxide; MACEs, major adverse clinical events.

In patients with DM, the risk of MACEs was significantly higher in those with TMAO levels above 371.59 ng/mL (HR = 1.744, 95% CI 1.084- 2.808, *p* = 0.022). However, in non-DM patients, neither continuous nor categorical variables were associated with independent risk for MACEs (*p* > 0.05) (**Table 2**). Kaplan–Meier analysis showed that the cumulative MACE event rate. As evidenced by the Kaplan–Meier analysis in **Figure 4**, CAD patients in tertile 3 group (TMAO levels >318.28 ng/mL) experienced a cumulative event rate for MACEs of 12.7% (266 out of 2090) after 54 months of follow-up. As shown in **Supplementary Figures 2A, B**, the risk of MACEs was higher in DM patients with higher TMAO levels (> 371.59 ng/mL) and in non-DM patients with higher TMAO levels (> 265.28 ng/mL).

The median TMAO level of 223.83 ng/mL was used as the cut-off value, and further analysis of MACEs according to TMAO levels and DM were analyzed. Kaplan–Meier curve analysis showed that patients with both high TMAO levels (>223.83 ng/mL) and DM had a markedly higher cumulative incidence of MACEs than patients with low TMAO levels (<223.83 ng/mL) and non-DM patients (log-rank *p* < 0.001), as shown in **Supplementary Figure 3**.

### 3.5 Subgroup analysis

Next, we conducted the subgroups analysis stratified by age, sex, BMI, hypertension, hyperlipidemia, DM, and stroke in CAD patients. As shown in **Supplementary Figure 4**. TMAO was associated with MACEs in different subgroups. The results indicated a significant interaction between DM subgroups and TMAO on the risk of MACEs (*p* for interaction<0.05). But no interaction was found in other groups. Additionally, we performed stratified analyses on patients with and without diabetes, including factors such as age, gender, BMI, hypertension, hyperlipidemia, and stroke. Intuitive forest plots were created to illustrate the impact of TMAO on MACEs under different stratification conditions, as shown in **Supplementary Figures 5**, **6**. In patients with and without diabetes, no interaction was found between TMAO levels and age, gender, BMI, hypertension, hyperlipidemia, or stroke(*p* for interaction > 0.05).

In addition, We conducted sensitivity analysis on the primary composite endpoint and found that, compared to the Tertile1 group, patients with high TMAO levels (Tertile 3 group)had a higher risk of stroke (HR=1.921, 95% CI 1.005-3.499, *p*=0.033) and all-cause mortality (HR=3.128, 95% CI 2.075-4.715, *p* < 0.001). However, statistical significance was not observed in terms of nonfatal myocardial infarction.

## 4 Discussion

In this cohort study, we discovered several important findings. First, we observed that CAD patients with high levels of TMAO in their bloodstream were more likely to experience MACEs. The positive correlation between peripheral TMAO levels and adverse prognosis has been previously reported in the general population, but our study specifically explored this relationship in CAD patients with DM. We found that people with DM had higher TMAO levels than those without DM. In CAD patients with DM, but not those without DM, high TMAO levels were linked to an increased risk of MACEs, even following the adjustment for traditional risk factors. Furthermore, a nonlinear relationship and threshold effects of TMAO were found for MACEs in CAD and CAD-DM patients, with TMAO turning points of 318.28 ng/mL and 371.59 ng/mL, respectively.

Existing studies have revealed that TMAO is an independent risk factor for cardiovascular disease, and experts such as Ringel et al. have found that TMAO levels can predict long-term mortality in patients with suspected CAD (14). Kuo et al. (15) revealed a positive association between serum TMAO level and MetS among patients with CAD. Matsuzawa et al. (16) have also researched the subject and found that chronic increases in TMAO levels can independently predict future cardiovascular events. Through our research, we discovered that patients with CAD who have high levels of TMAO are more likely to develop MACEs. This finding is consistent with previous studies on the subject. Additionally, our study revealed a threshold effect, indicating a nonlinear correlation between TMAO concentration and MACEs, with a clear dose-response relationship. Hence, it is conceivable that higher TMAO concentrations correlate with increased risks of MACE in populations with CAD. TMAO is a metabolite produced by the intestinal flora, and it has gained significant attention in recent years due to its potential role in cardiovascular disease (17). It has a complex impact on cardiovascular disease. It contributes to the development of atherosclerosis through its effects on cholesterol and sterol metabolism and enhances macrophage cholesterol aggregation. TMAO also promotes inflammation (18, 19), causes endothelial cell dysfunction (20), increases platelet activation, and stimulates thrombosis (21, 22), all of which are key pathophysiological mechanisms of cardiovascular adverse events linked to TMAO. Overall, our study again highlights the importance of monitoring TMAO levels in patients with CAD to mitigate the risk of MACEs.

The prevalence of DM has become a major concern for public health. Cardiovascular disease is the most prevalent and perilous complication that can arise from diabetes, often resulting in mortality for those who suffer from this condition (20, 23). The microbiota regulates the levels of bioactive metabolites in the plasma, acting as a new endocrine organ (24). Studies show that diet and gut microbiota play a crucial role in the initiation and progression of DM (25). Numerous studies conducted on both animals and humans have shown that the gut microbiota has a crucial impact on the overall metabolism of the host and is linked with cardiometabolic diseases, such as DM (26–28). People with both DM and CAD experience notable changes in their gut microbiota, including reduced diversity and functional differences in the metagenome (29). Studies have shown that alterations in diet and gut microbiota, rather than genetics, have a notable effect on TMAO levels in both mice and humans (29). Studies on animals have revealed that diabetic db/db mice have TMAO levels 10 times higher than nondiabetic db/db mice (30). We found similar results in our study on humans. DM patients had higher TMAO levels (264.89 ng/mL) than non-DM patients (192.66 ng/mL).

Li et al. (31) found that higher serum TMAO was associated with a higher risk of type-2 diabetes and an increase in fasting glucose among middle-aged and older Chinese adults. A recent meta-analysis showed a positive dose-dependent relationship between the levels of circulating TMAO and an increased risk of type-2 diabetes mellitus (9). Previous research has shown that higher plasma levels of TMAO are associated with stroke severity and has suggested a link between plasma levels of TMAO and glycemic changes in diabetes patients with acute ischemic stroke. To date, few studies have reported the association between TMAO levels and prognosis in DM patients. Our study found that in CAD patients with DM, rather than non-DM patients, TMAO levels independently predicted poor prognosis during a maximum 6-year follow-up.

The relationship between TMAO and DM seems to be interactive. A recent study found that TMAO levels in human beings may be associated with higher glucose and lipids, circulating TMAO may be potential biomarkers of interest in the development of type 2 diabetes (32). In addition, in mice fed a high-fat diet, adding TMAO worsens glucose tolerance, obstructs hepatic insulin signaling, and causes inflammation in adipose tissue. Moreover, a decrease in choline, a precursor of TMAO, was significantly linked with a 2-year enhancement in glucose and insulin resistance (10). An increase in TMAO and related metabolites can negatively impact glucose and insulin resistance, potentially contributing to the development of DM. The link between TMAO and insulin resistance may be due to increased N-nitroso compounds (NOCs). These compounds were shown to be associated with the development of insulin-resistant conditions such as DM (33). Insulin resistance and subsequently increased insulin levels are linked to heightened FMO3 activity, which in turn leads to elevated levels of TMAO (34). When TMAO levels in the bloodstream are high, there is a reduction in the expression of enzymes and proteins involved in bile acid synthesis and transport, which results in a decrease in the overall bile acid pool size (35). A smaller bile acid pool size may lead to obesity and diabetes due to inflammation and reduced energy expenditure (36). In turn, imbalances in gut bacteria among diabetes patients may affect TMAO levels. High blood sugar levels can trigger the overexpression of FMO3, an enzyme involved in the TMAO pathway, which can lead to increased TMAO levels and worsen atherosclerosis (32). It seems to be a vicious circle.

Our study has several limitations. Firstly, the influence of dietary factors on TMAO levels, such as dietary intake from foods like fish, has not been taken into account, and antibiotic use may have influenced TMAO levels, and our study did not include antibiotic use, which could introduce bias. Our study specifically targeted the measurement of TMAO levels, thus we lack data on upstream metabolites such as carnitine, choline, and betaine. Second, this study only measured baseline TMAO levels and did not consider the effect of TMAO variation on MACEs, which needs to be confirmed in future studies. In addition, we were unable to collect data on glucose-lowering medications and information reflecting the severity of the lesions, among other things, which may have biased the results. And further studies are also required to determine whether intervention can reduce the risk of adverse clinical outcomes in CAD patients.

## 5 Conclusions

In conclusion, our study highlighted the association between elevated TMAO levels and adverse outcomes in CAD patients, especially combined with DM. The measurement of TMAO level could help determine the risk for secondary prevention among CAD patients with DM comorbidity.

## Data availability statement

The original contributions presented in the study are included in the article/**Supplementary Material**. Further inquiries can be directed to the corresponding author.

## Ethics statement

The studies involving humans were approved by Beijing Hospital, National Center of Gerontology, Institute of Geriatric Medicine (approval number 2016BJYYEC-121-02). The studies were conducted in accordance with the local legislation and institutional requirements. The participants provided their written informed consent to participate in this study. Written informed consent was obtained from the individual(s) for the publication of any potentially identifiable images or data included in this article.

## Author contributions

XY: Data curation, Formal analysis, Methodology, Resources, Visualization, Writing – original draft, Writing – review & editing. YW: Data curation, Formal analysis, Visualization, Writing – original draft. RY: Data curation, Formal analysis, Resources, Writing – original draft. ZW: Data curation, Formal analysis, Methodology, Visualization, Writing – original draft. XW: Data curation, Methodology, Project administration, Resources, Writing – review & editing. SW: Data curation, Formal analysis, Resources, Software, Writing – review & editing. WZ: Data curation, Methodology, Project administration, Resources, Writing – review & editing. JD: Conceptualization, Data curation, Funding acquisition, Investigation, Validation, Visualization, Writing – review & editing. WC: Conceptualization, Data curation, Funding acquisition, Investigation, Resources, Supervision, Validation, Writing – review & editing. FJ: Conceptualization, Funding acquisition, Investigation, Resources, Supervision, Validation, Writing – review & editing. WG: Conceptualization, Data curation, Investigation, Supervision, Validation, Writing – review & editing.
