# Supplementary material for: Trimethylamine N-oxide predicts cardiovascular events in coronary artery disease patients with diabetes mellitus: a prospective cohort study
Source: Front Endocrinol (Lausanne). 2024 Jul 18;15:1360861. doi: 10.3389/fendo.2024.1360861 (PMC11291261; doi:10.3389/fendo.2024.1360861)
Supplement: Supplementary file 1 [file DataSheet_1.docx]

**Supplemental Figure 1** Patient flow chart for the study cohort.


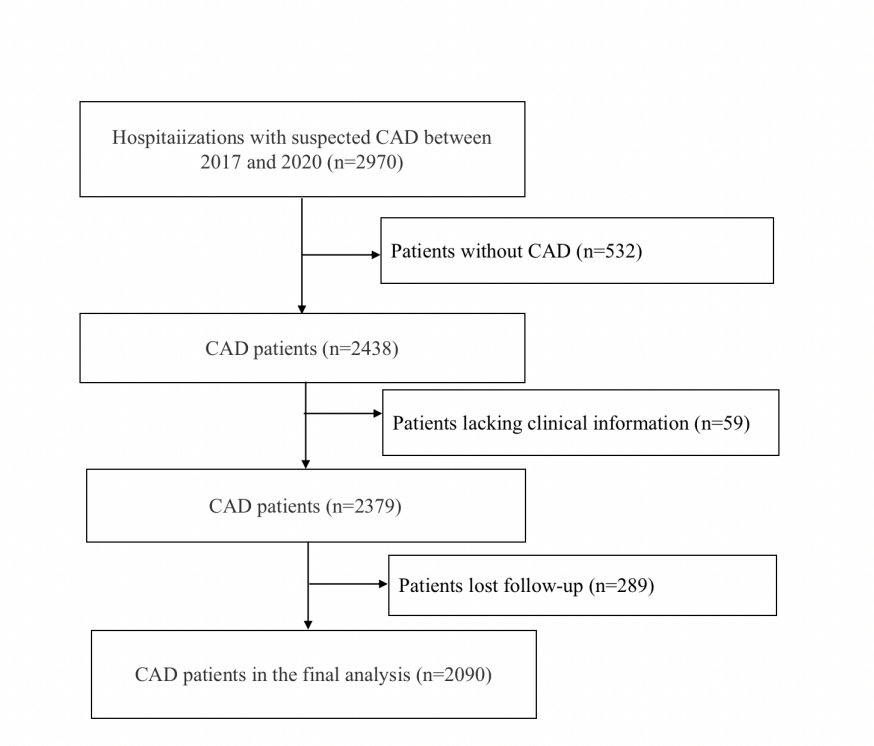


**Supplemental Figure 2** Kaplan–Meier curves for MACEs according to TMAO levels in patients with DM (A) and with non-DM (B). DM, diabetes mellitus; TMAO, trimethylamine N-oxide; MACEs, major adverse clinical events.

A


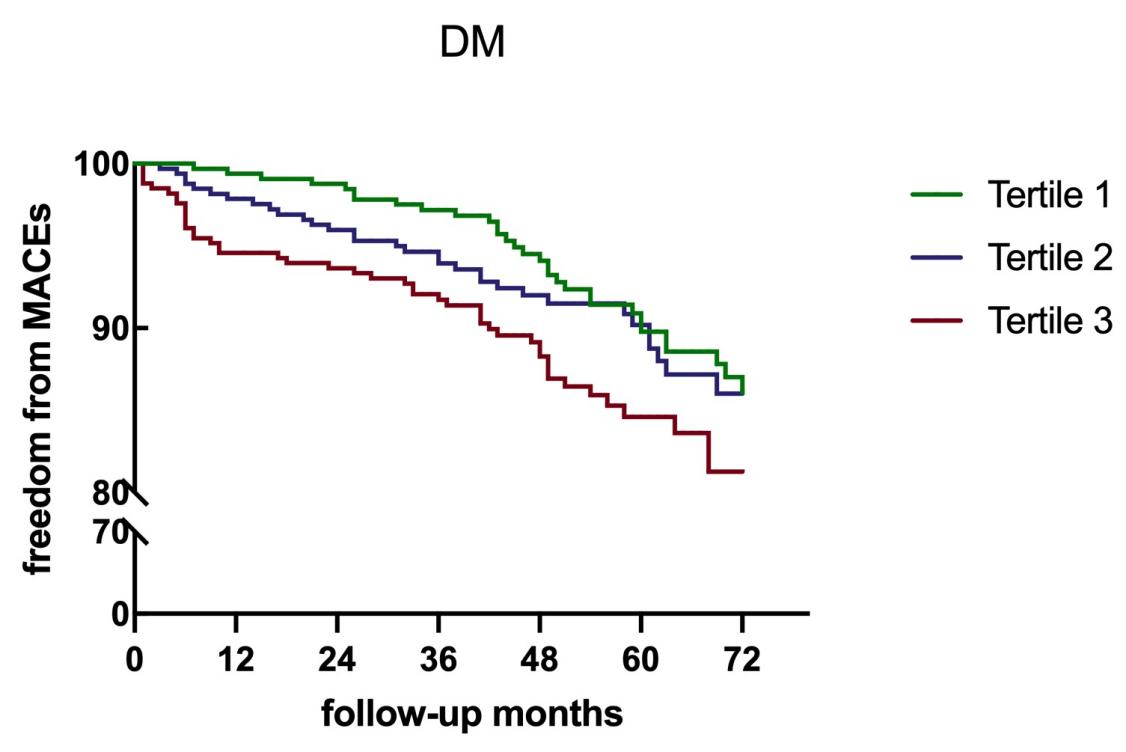


Log-rank *p* value =0.020

**B**

**
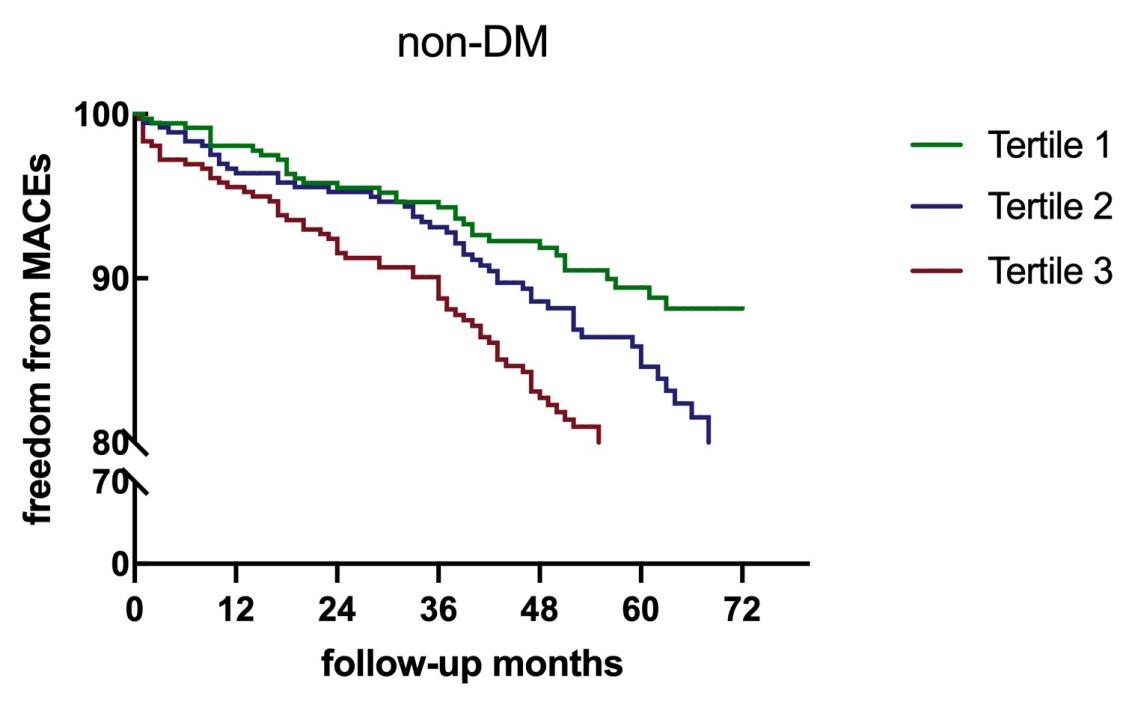
**

Log-rank *p* value <0.001

**Supplemental Figure 3** Clinical outcomes according to the combination of TMAO concentration (< 223.83 ng/mL or ≥ 223.83 ng/mL) and whether combined with DM (DM or non-DM). TMAO, trimethylamine N-oxide; DM, diabetes mellitus; MACEs, major adverse clinical events.


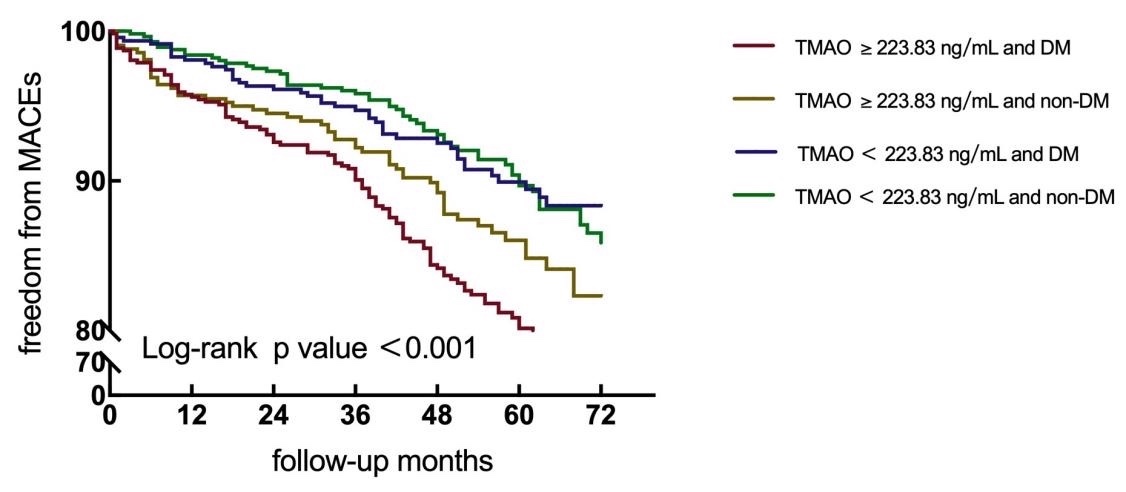


**Supplemental Figure 4** Association between TMAO (per SD increase) and MACEs in different subgroups of patients with CAD. HR, hazard ratio; CI, Confidence interval; BMI, body mass index; DM, diabetes mellitus; CAD,coronary artery disease. orizontal axis: Hazard Ratio (HR) for occurrence of MACEs

**
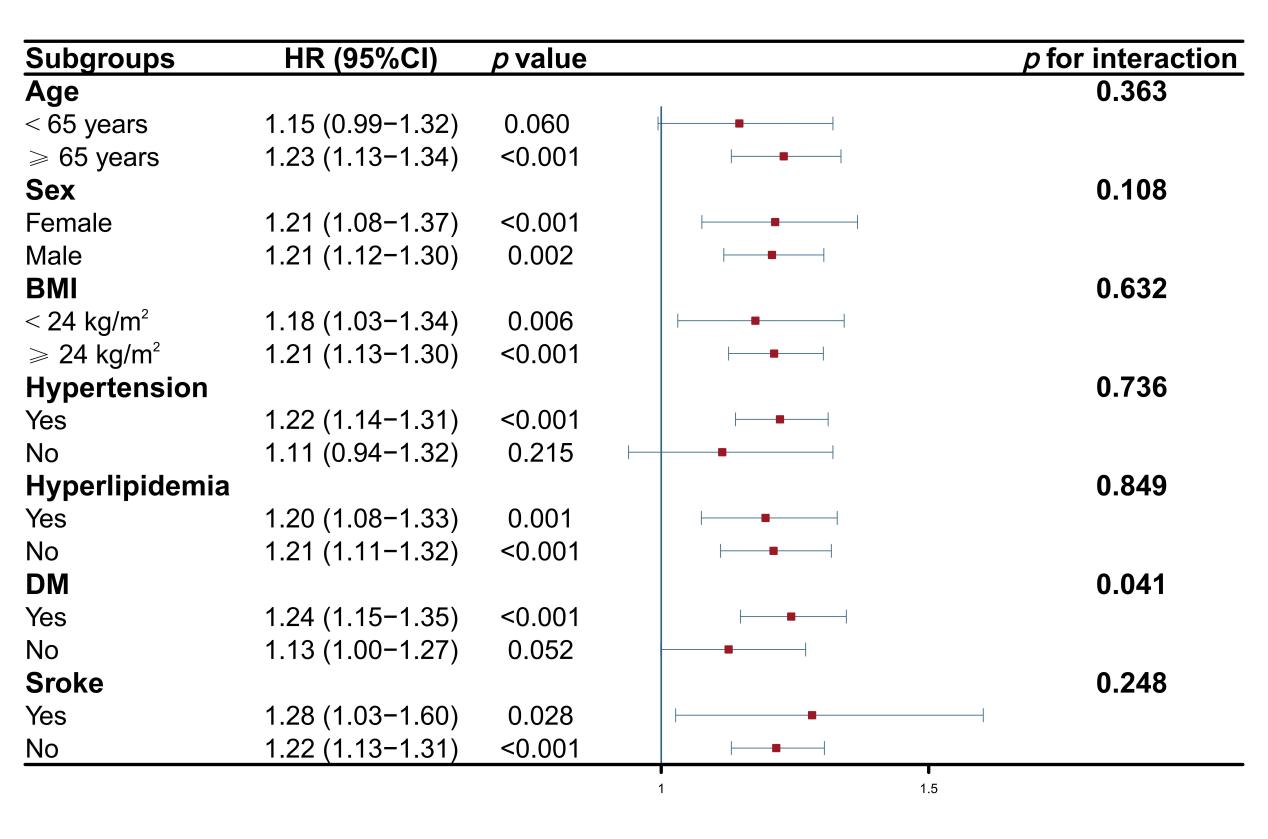
**

**Supplemental Figure 5** Association between TMAO (per SD increase) and MACEs in different subgroups of patients with CAD and DM. HR, hazard ratio; CI, Confidence interval; BMI, body mass index; DM, diabetes mellitus. orizontal axis: Hazard Ratio (HR) for occurrence of MACEs

**
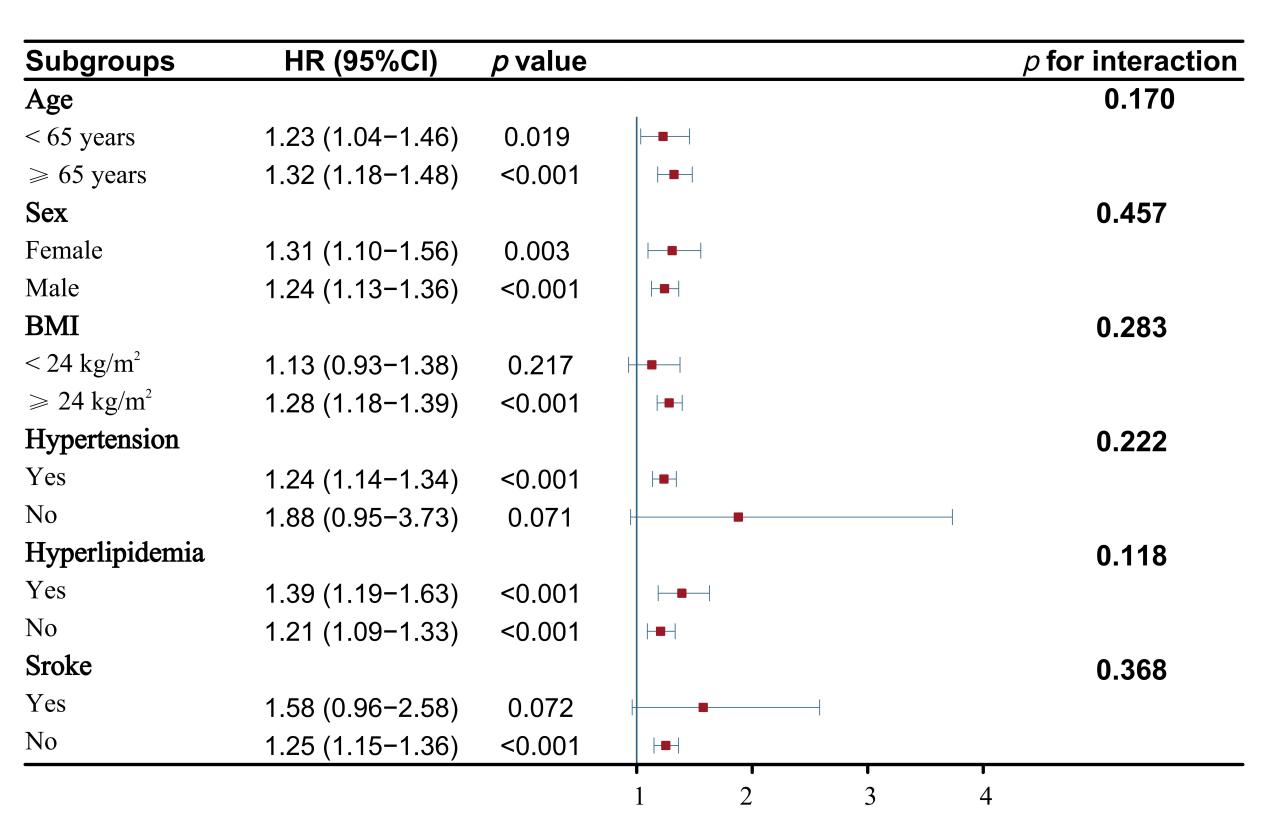
**

**Supplemental Figure 6** Association between TMAO (per SD increase) and MACEs in different subgroups of patients with CAD and non-DM. HR, hazard ratio; CI, Confidence interval; BMI, body mass index; DM, diabetes mellitus. orizontal axis: Hazard Ratio (HR) for occurrence of MACEs


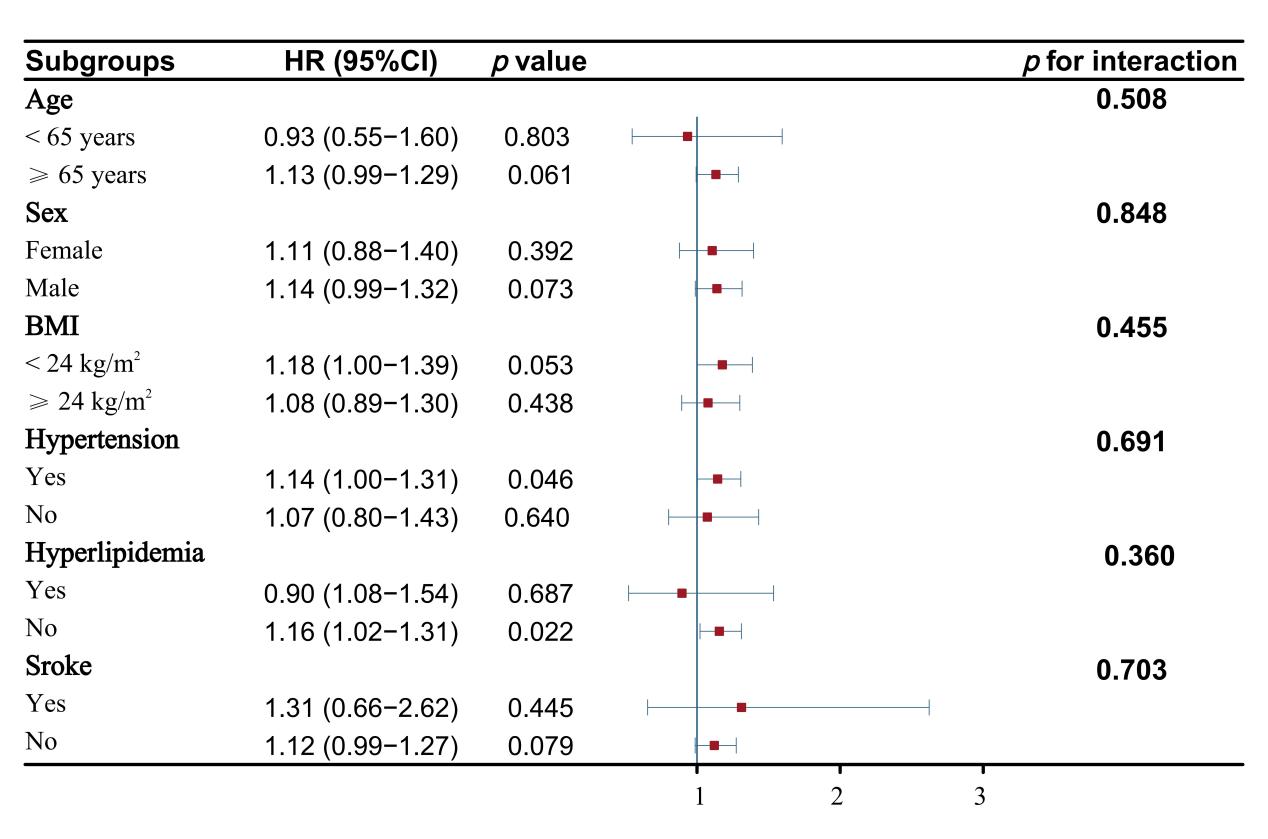


**Supplemental Table 1.**Baseline clinical characteristics based on MACEs.

| Variables | All(n=2090) | MACEs group  (n=266) | MACEs-free group (n=1824) | *p-*value |
| --- | --- | --- | --- | --- |
| age, years | 66.2±10.8 | 70.3±11.3 | 65.6±10.5 | <0.001 |
| male, % | 1392(66.6%) | 175(65.8%) | 1217(66.7%) | 0.763 |
| BMI, kg/m^2^ | 26.2±6.7 | 25.5±7.8 | 26.3±6.5 | 0.069 |
| current smoking, % | 703(33.6%) | 88(33.1%) | 615(33.9%) | 0.792 |
| hypertention, % | 1654(79.1%) | 214(80.5%) | 1440(78.9%) | 0.573 |
| hyperlipidemia, % | 976(46.7%) | 112(42.1%) | 864(47.4%) | 0.150 |
| DM, % | 1094(52.3%) | 156(58.6%) | 938(51.4%) | 0.028 |
| stroke, % | 241(11.5%） | 59(22.2%) | 182(10.0%) | <0.001 |
| history of statin use, % | 773(37.0%) | 98(41.9%) | 675(41.4%) | 0.886 |
| family history of premature CAD, % | 164(7.8%) | 19(7.1%) | 145(7.9%) | 0.455 |
| prior PCI, % | 801(38.3%) | 109(41.0%) | 692(37.9%) | 0.175 |
| Laboratory test |  |  |  |  |
| TC, mmol/L | 0.77±0.97 | 3.57±0.84 | 3.53±0.80 | 0.511 |
| TG, mmol/L | 3.54±0.80 | 1.63±0.86 | 1.78±0.99 | 0.018 |
| HDL-C, mmol/L | 1.00±0.26 | 0.99±0.26 | 1.00±0.26 | 0.514 |
| LDL-C, mmol/L | 2.02±0.70 | 2.09±0.73 | 2.01±0.70 | 0.099 |
| FBG, mmol/L | 7.1±2.7 | 7.2±2.9 | 7.1±2.7 | 0.327 |
| ALT | 19.60(14.80,27.70) | 17.95(13.90,26.10) | 19.90(14.90,27.90) | 0.015 |
| AST | 20.40(17.60,25.10) | 20.90(17.70,26.03) | 20.40(17.60,25.00) | 0.628 |
| Crea,umol/L | 78.00(68.00,89.00) | 85.00(69.00,100.00) | 77.00(68.00,88.00) |  |
| TMAO, ng/ml | 223.83(132.43,383.58) | 307.0(157.88,510.45) | 216.30(129.08,370.95) | <0.001 |
| Medication at discharge |  |  |  |  |
| Aspirin,% | 1831(87.6%) | 232(87.2%) | 1599(87.7%) | 0.560 |
| Clopidogrel,% | 1435(68.7%) | 197(74.1%) | 1238(67.9%) | 0.063 |
| Ticagrelor,% | 41(2.0%) | 2(0.8%) | 39(2.1%) | 0.178 |
| ACEI/ARB, % | 886(42.4%) | 123(46.2%) | 763(41.8%) | 0.213 |
| β-bloker, % | 1176(56.3%) | 149(56.0%) | 1027(56.3%) | 0.563 |

Abbreviation: BMI, body mass index; DM, diabetes mellitus; CAD, coronary artery disease; PCI, percutaneous coronary intervention; TC,total cholesterol; TG,triglycerides; HDL-C, high-density lipoprotein cholesterol; LDL-C, low-density lipoprotein cholesterol; ALT, alanine aminotransferase; AST, aspartate aminotransferase; TMAO, trimethylamine N-oxide; ACEI, angiotensin converting enzyme inhibitors; ARB, angiotensin receptor blocker.

**Supplemental Table 2.**Baseline clinical characteristics based on DM.

| Variables | non-DM（n=1094） | DM（n=996） | *p-*value |
| --- | --- | --- | --- |
| Age，years | 65.6±11.1 | 66.7±10.4 | 0.028 |
| Male gender | 671(67.4%) | 721(65.9%) | 0.478 |
| BMI，kg/m^2^ | 25.9±6.5 | 26.4±6.8 | 0.071 |
| current smoking,% | 341(34.4%) | 362(33.2%) | 0.553 |
| hypertention,% | 747(75.0%) | 907(82.9%) | ＜0.001 |
| hyperlipidemia,% | 439(44.1%) | 537(49.1%) | 0.067 |
| Stoke,% | 109(10.9%) | 132(12.1%) | 0.422 |
| History of statin use,% | 344(34.5%) | 429(39.2%) | 0.021 |
| Family history of premature CAD,% | 78(7.8%) | 86(7.9%) | 0.747 |
| Laboratory test |  | | |
| TC,mmol/L | 1.73±0.96 | 1.80±0.98 | 0.082 |
| TG,mmol/L | 3.54±0.78 | 3.53±0.82 | 0.863 |
| HDL-C,mmol/L | 1.02±0.26 | 0.98±0.25 | ＜0.001 |
| LDL-C,mmol/L | 2.02±0.70 | 2.03±0.71 | 0.812 |
| FBG,mmol/L | 5.3(4.9,5.9) | 7.9(6.5,10.0) | ＜0.001 |
| Crea,umol/L | 79(69,88) | 77(67,91) | 0.364 |
| TMAO,ng/ml | 192.66(122.54,307.68) | 264.89(144.66,442.40) | ＜0.001 |
| Medication at discharge |  | | |
| Aspirin,% | 852(85.5%) | 979(89.5%) | 0.018 |
| Clopidogrel,% | 623(62.6%) | 812(74.2%) | ＜0.001 |
| Ticagrelor,% | 25(2.5%) | 16(1.5%) | 0.201 |
| ACEI/ARB,% | 351(35.2%) | 535(48.9%) | ＜0.001 |
| β-bloker,% | 526(52.8%) | 650(59.4%) | 0.008 |

Abbreviation: BMI, body mass index; DM, diabetes mellitus; CAD, coronary artery disease; TC,total cholesterol; TG,triglycerides; HDL-C, high-density lipoprotein cholesterol; LDL-C, low-density lipoprotein cholesterol; TMAO, trimethylamine N-oxide; ACEI, angiotensin converting enzyme inhibitors; ARB, angiotensin receptor blocker.
